# Bacterial isolates, antibiotic susceptibility, and determinants of positive blood culture in children with community-acquired pneumonia in Central Tanzania

**DOI:** 10.11604/pamj.2025.52.145.46769

**Published:** 2025-12-05

**Authors:** Rehema Tagalile, Fransisca Kimaro, Emmanuel Nkuwi, Dina Mahamba

**Affiliations:** 1 Department of Paediatrics and Child Health, University of Dodoma Hospital, Dodoma, Tanzania,; 2 Department of Peadiatrics and Child Health, School of Clinical Medicine, Muhimbili University of Health and Allied Sciences, Dar es Salaam, Tanzania,; 3 Department of Microbiology and Parasitology, School of Medicine and Dentistry, The University of Dodoma, Dodoma, Tanzania,; 4 Department of Paediatrics and Child Health, School of Medicine and Dentistry, The University of Dodoma, Dodoma, Tanzania

**Keywords:** Bacteremia, community-acquired, pneumonia, children, antibiotic, predictors

## Abstract

**Introduction:**

despite the widespread coverage of Haemophilus influenzae type b (Hib) and pneumococcal conjugate vaccines, the mortality of children under five years due to community-acquired pneumonia (CAP) in Tanzania remains unacceptably high. This study aimed to identify bacterial isolates, antibiotic susceptibility, and factors associated with blood culture positivity in children under five with CAP.

**Methods:**

a cross-sectional study involving 195 children under-five years of age who were clinically diagnosed with CAP was conducted at a referral hospital in central Tanzania from October 2018 to March 2019. Blood samples were taken, identification of culture isolates was performed through conventional bacteriological methods, followed by antibiotic susceptibility testing. Demographic and other data were collected using a standardized tool.

**Results:**

bacteremia was prevalent in 7.2% of participants, with Staphylococcus aureus being the most common isolate (57.1%). Overall, isolates were less sensitive to the WHO first-line antibiotics recommended for CAP treatment (penicillin and cephalosporins) but were sensitive to ciprofloxacin and clindamycin. Fever (>38.5°C), oxygen saturation (<90%), the need for oxygen therapy, a high respiratory rate (>60 cycles/minute), and leucocytosis (>15,000 cells/μl) were independent factors associated with positive blood culture.

**Conclusion:**

bacteraemia in children with CAP was nearly 10%, predicted by high respiratory rates, hypoxia, fever, and leucocytosis. Most of the isolates were insensitive to antimicrobials listed in the WHO-recommended first-line treatment for CAP, outlining the importance of routine culture and antimicrobial susceptibility.

## Introduction

Community-acquired pneumonia (CAP) refers to a lower respiratory tract infection occurring in a person who has not resided in a hospital or health care facility in the preceding 14 days [1]. Overall, CAP accounts for a significant proportion of childhood pneumonia cases, causing morbidity and mortality in developing countries and globally [2-4]. Despite efforts to prevent CAP, including widespread vaccination, Tanzania remains among the low- and middle-income countries (LMICs) with the highest number of CAP-related deaths [5,6]. The persistence of CAP cases is likely due to pathogens not covered by the Pneumococcal Conjugated Vaccine (PCV) and *Haemophilus influenzae* type b (Hib) vaccine.

Measures to prevent and treat CAP in children face multiple challenges, including accurate clinical diagnosis and determination of etiology [7]. The symptoms of CAP differ by age and can be nonspecific in pediatric patients, with causative agents often varying based on age and geographical region. Overall, viruses account for more than 50% of CAP cases in children under five years, particularly during the first two years of life [3]. *Streptococcus pneumonia* is the most common bacterial cause, followed by *Haemophilus influenzae, Streptococcus pyogenes, Staphylococcus aureus, Moraxella catarrhalis, Mycoplasma pneumoniae, Chlamydia trachomatis*, and *Ureaplasma urealyticum*. On several occasions, these pathogens have shown reduced sensitivity to amoxicillin and cephalosporin, the WHO-recommended first-line antibiotics for CAP treatment [7,8].

Despite CAP being the most important cause of morbidity among children under five in the developing world, bacterial isolates, antibiotic susceptibility, and factors associated with positive blood cultures have not been adequately studied, especially in the context of expanded vaccine coverage. This has resulted in undocumented patterns of etiology, a lack of evidence-based prevention strategies, and the irrational use of antibiotics, thereby exacerbating the global burden of antimicrobial resistance. This study was therefore conducted to determine the bacterial isolates, antibiotic susceptibility, and factors associated with positive blood cultures among children with CAP in central Tanzania.

## Methods

**Study design, setting, and participants:** we conducted a hospital-based cross-sectional analytical study from October 2018 to March 2019 at Dodoma Regional Referral Hospital (DRRH) in central Tanzania. Dodoma Regional Referral Hospital (DRRH) is an affiliated teaching hospital for the University of Dodoma with a total bed capacity of 434, including 54 beds for paediatric patients. Approximately 300 paediatric patients are admitted per month. The study enrolled all children aged 2 to 59 months who were admitted with signs and symptoms of CAP, provided that their parents or guardians had signed written informed consent. The minimum sample size of 195 children was calculated using the Leslie Kish formula, based on an 8% prevalence of bacteraemia among children with CAP reported by Sigaúque *et al*. [9], with a 5% margin of error and 95% confidence interval. Children with a history of foreign body aspiration, chemical ingestion, and who had a history of hospital visits or admissions within the past two weeks were excluded. Children meeting eligibility criteria were conveniently sampled until the minimum sample size was attained.

**Data collection methods and tools:** a structured questionnaire was used to collect sociodemographic data of both children and parents or guardians (age, sex, marital status, education level, occupation, family history of smoking, and size of family). Additionally, clinical information on the children, such as cough, difficulty breathing, runny nose, fever, nutritional status, and vaccination status, was collected.

In particular, information on receiving pneumococcal conjugate and *Haemophilus influenzae* type b vaccines was gathered from the Reproductive and Child Health (RCH) card. A thorough physical examination was then performed while looking for signs of CAP and nutritional status, followed by blood sample collection for culture and sensitivity testing. All the children were managed according to the respective standard hospital guidelines [10]. Operationally, we defined CAP as cough and/or difficulty in breathing and any of the added symptoms, including increased respiratory rate, respiratory distress (grunting, head nodding, lower chest wall indrawing), and oxygen saturation <90% in room air for a child who had no history of hospital visit or admission within past two weeks before current admission [7]. Malnutrition was diagnosed based on WHO growth standards, defined as mid-upper arm circumference (MUAC) < 11.5 cm in children aged 6 months and above, or weight-for-height/length Z-score <-2 standard deviations (SD), and/or the presence of bilateral pitting edema [11].

**Laboratory procedures:** for blood culture and sensitivity testing, 3 mL of venous blood was aseptically drawn prior to the initiation of antibiotic therapy, following sterilization of the puncture site with 70% isopropyl alcohol. The drawn blood was then inoculated directly into blood culture bottles (BD Bactec Peds Plus, Mississauga, Canada), which were incubated in an automated blood culture machine (BD Bactec 9050, Illinois, USA). Blood samples were incubated and periodically monitored for turbidity.

Positive blood culture samples were Gram-stained and subcultured on 5% CO_2_ blood, MacConkey, and chocolate agar plates. The bacterial isolates were identified using conventional biochemical tests, and antibiotic sensitivity testing was performed using the disc diffusion method in accordance with the Clinical Laboratory Standard Institute (CLSI) guidelines [12]. The antibiotics used for susceptibility testing were ampicillin, chloramphenicol, ceftriaxone, ciprofloxacin, gentamicin, erythromycin, clindamycin, cefuroxime, and penicillin. *Staphylococcus aureus* (ATC 25923) and *Escherichia coli* (ATCC 25922) strains were used to ensure quality control.

**Data analysis:** binary logistic regression was used to identify factors independently associated with positive blood culture results (outcome: positive versus negative). Variables with p<0.0.5 in univariate analysis were included in the multivariate model to adjust for confounders. Independent variables assessed included age, sex, nutritional status, fever, hypoxia, prior antibiotic use, existing chronic disease, oxygen saturation during admission, and white blood cell count. Results are reported as odds ratios (ORs) with 95% confidence intervals (CIs), and p<0.05 was considered significant. Analyses were performed using SPSS version 25.

**Ethics approval and consent to participate:** ethical clearance was sought from the University of Dodoma research ethics and scientific review committee (reference number UDOM/DRP/134 VOL VI/66-14). Permission to conduct the study was obtained from the Dodoma Regional Referral Hospital authorities. Informed consent was obtained from all parents and guardians who agreed that their children would participate.

## Results

**Figure 1:**
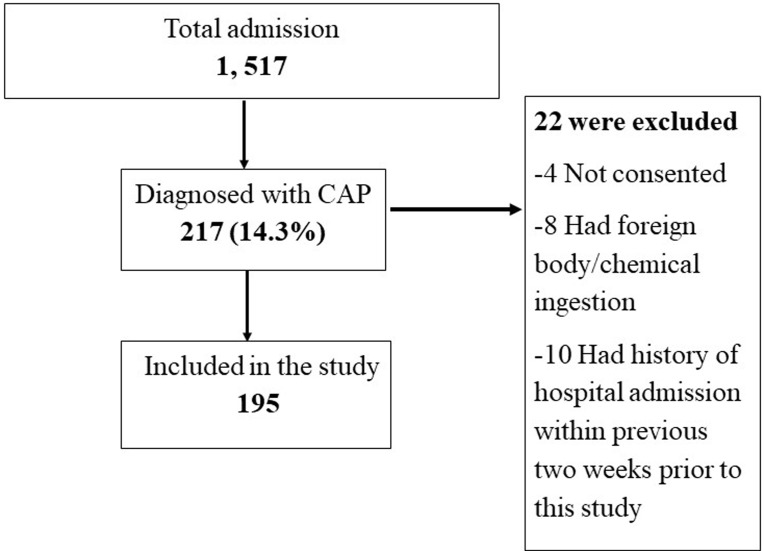
enrolment process of the 195 children diagnosed with CAP in Dodoma, Central Tanzania, from October 2018 to March 2019

**Study participants’ enrolment:** during the study duration, a total of 1,517 children were admitted to DRRH. CAP was clinically diagnosed among 14.3% (217) of participants, from which 195 children passed the eligibility criteria for the study (Figure 1).

**Sociodemographic characteristics of the enrolled children and their parents or guardians:** a total of 195 children were clinically diagnosed with CAP and enrolled in this study. The study participants had a mean age of 14.65± SD months, with the majority (62%) being males (Table 1). Of note, most parents or guardians (70.3%) had a primary school education, and 13.8% reported having a family member who smoked cigarettes. Additionally, nearly forty percent (39.5%) of the children had a history of antibiotic use for an average of two (2) days within the previous 14 days (Table 1).

**Table 1:** sociodemographic characteristics of the parents or guardians and their 195 children diagnosed with CAP in Dodoma, Central Tanzania, from October 2018 to March 2019

Variable	Frequency (n)	Percentage (%)
**Age of the children (months)**		
2 - 12	118	60.5
13 - 24	43	22.1
≥25	34	17.4
**Sex**		
Male	129	66.2
Female	66	33.8
**Age of parents/guardian (years)**		
<20	7	3.6
20 - 30	119	61.0
31 - 40	61	31.3
>40	8	4.1
**Education of the parent/guardian**		
Informal	20	10.3
Primary school	137	70.3
Secondary school	28	14.4
University/College	10	5.1
**Cigarette smoking (family member)**		
Yes	27	13.8
No	168	86.2
**History of antibiotic use**		
Yes	77	39.5
No	118	60.5

**Figure 2:**
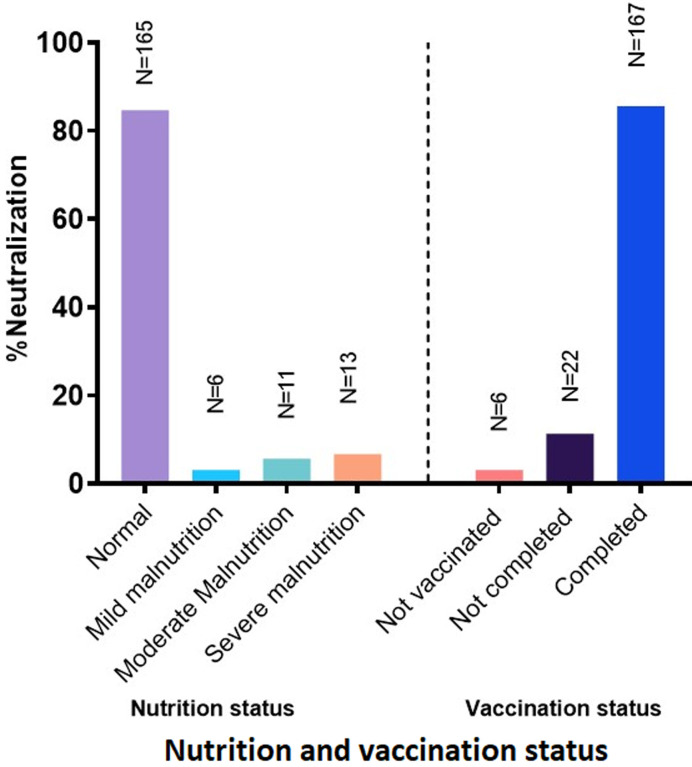
nutritional and vaccination status of 195 children diagnosed with CAP in Dodoma Region, Central Tanzania, from October 2018 to March 2019

**Nutritional and vaccination status of children with CAP:** among all the children enrolled, 6.7% (13/195) were severely malnourished, followed by 5.6% (11/195) who were moderately malnourished. On the other hand, 85.6% (167/195) were completely immunized, whereas 11.3% (24/195) were partially immunized (had only up to 2 vaccine doses), and 3.1% (6/195) were not immunized. In particular, the majority (96.9%, 189/195) of the children had received at least a single dose of Pneumococcal Conjugated Vaccine (PCV) and *Haemophilus influenzae* type B (HiB) vaccine (Figure 2).

**Figure 3:**
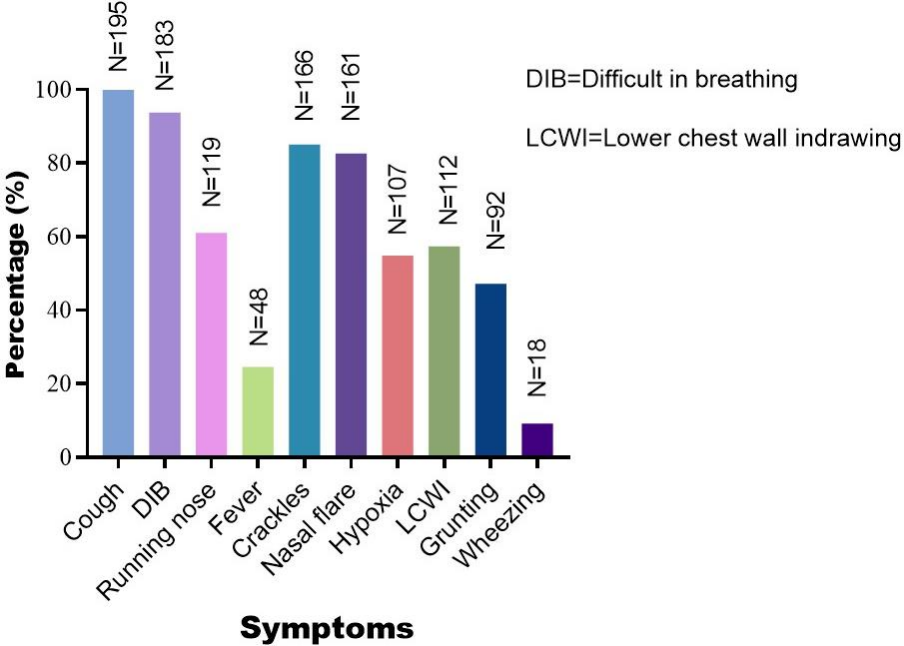
clinical characteristics of 195 children diagnosed with CAP in Dodoma, Central Tanzania, from October 2018 to March 2019

**Clinical characteristics of children with CAP:** cough was the most common clinical presentation observed in all the children (100%), followed by difficulty in breathing, which was noted in 93.8% (183/195) of the participants (Figure 3). Slightly more than half of the children presented symptoms such as a running nose, crackles, nasal flares, hypoxia, and lower chest wall indrawing. Fever was present in a quarter of the participants, whereas wheezing was observed in only 9.2% of all children with a CAP diagnosis (Figure 3). None of the children presented with signs of complicated pneumonia, and no participant required ICU admission or intubation during the study period.

**Figure 4:**
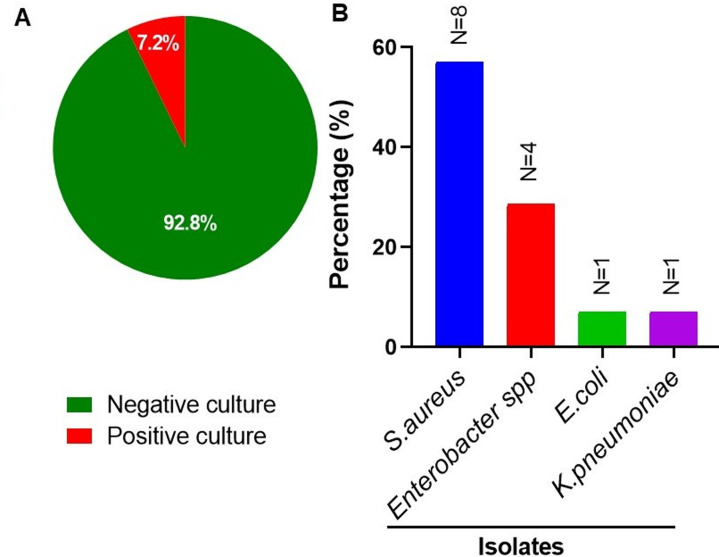
blood culture results (A) and distribution of bacterial isolates (B) detected in the blood samples obtained from 195 children diagnosed with CAP in Dodoma, Central Tanzania, from October 2018 to March 2019

**Prevalence and distribution of bacterial isolates from positive blood cultures:** among the 195 children diagnosed with CAP, 7.2% (14/195) had positive blood cultures (Figure 4A). *Staphylococcus aureus* was the most frequently isolated pathogen, accounting for 57.1% (8/14) of the positive cultures, followed by *Enterobacter spp*., which comprised 28.6% (4/14) of the isolates. *Klebsiella pneumonia* and *Escherichia coli* were each identified as a single isolate, representing 7.1% (1/14) of the positive cultures (Figure 4B).

**Antibiotic susceptibility of isolates:** the isolates showed the highest sensitivity to ciprofloxacin, with 100% sensitivity observed for *Enterobacter spp*., *E. coli* and *K. pneumoniae spp*., whereas *S. aureus* exhibited 75% sensitivity. Clindamycin, on the other hand, performed best against *S. aureus* (100% sensitivity), while *K. pneumonia* displayed 75% sensitivity. Importantly, none of the isolates tested in this study were sensitive to the WHO-recommended first-line antibiotics for CAP (penicillin and cephalosporins) (Table 2).

**Table 2:** antibiotic sensitivity of the 15 bacterial isolates from 195 children diagnosed with CAP in Dodoma, Central Tanzania, from October 2018 to March 2019

Antibiotics	Sensitive isolates: n (%)			
	*S. aureus*	*Enterobacter sp*	*E. coli*	*K. pneumoniae*
Ampicillin	0 (0)	1 (25)	0 (0)	0 (0)
Chloramphenicol	2 (25)	2(50)	0 (0)	0 (0)
Ceftriaxone	3 (37.5)	2 (50)	0 (0)	0 (0)
Ciprofloxacin	6 (75)	4 (100)	1 (100)	1 (100)
Gentamicin	3 (37.5)	1 (25)	1 (100)	0 (0)
Erythromycin	2 (25)	0 (0)	0 (0)	0 (0)
Clindamycin	8 (100)	3 (75)	0 (0)	1 (100)
Cefuroxime	0 (0)	0 (0)	0 (0)	1 (100)
Penicillin	0 (0)	0 (0)	0 (0)	0 (0)

**Clinical and laboratory predictors for positive blood culture results:** multivariate logistic regression analysis revealed that fever (>38.5°C), oxygen saturation (<90%), the need for oxygen therapy, a high respiratory rate (>60 cycles/minute), and leucocytosis (>15,000 cells/μl) were strongly associated with bacteremia among children with CAP ((AOR 5.7 57) P=0.005), (AOR 2.7 95%CI [1.05 - 72.25] P = 0.015), (AOR 2.1 95%CI [1.41 - 7.676] P=0.022), (AOR 6.7 95%CI [2.018 - 22.47] P=0.002) and (AOR 8.895% CI [1.387 - 31.112] P=0.004) respectively) (Table 3).

**Table 3:** factors associated with positive blood cultures among 195 children diagnosed with CAP in Dodoma, Central Tanzania, from October 2018 to March 2019

	Univariate analysis		Multivariate analysis			
Variables	COR	95% CI	P	AOR	95% CI	P
**Fever**						
Yes	4.9	2.794 - 29. 658	0.001	5.7	1.016 - 31.57	0.005
No	1.00			1.00		
**LCWI**						
Yes	0.93	0.12 - 0.994	0.029	0.075	0.001 - 6.007	0.247
No	1.00			1.00		
**Respiratory rate (>60 cycles/min)**						
Yes	5.00	1.964 - 21.843	<0.001	6.7	2.018 - 22.47	0.002
No	1.00			1.00		
**Hypoxia**						
Yes	3.8	1.102 - 70.920	0.022	2.7	1.050 - 72.25	0.015
No	1.00			1.00		
**Given oxygen**						
Yes	1.901	1.12 - 7.009	0.005	2.1	1.41 - 7.676	0.022
No	1.00			1.00		
**Otitis media**						
Yes	0.206	0.037 - 0.830	0.046	1.193	0.83 - 17.085	0.897
No	1.00			1.00		
**WBC (cells/μl)**						
<10000	1.00			1.00		
10000 - 15000	1.276	0.468 - 16.812	0.118	1.576	0.030- 17.674	0.256
>15000	5.4	2.392 - 28.939	<0.001	8.8	1.387- 31.112	0.004

COR: crude odds ratio; CI: confidence interval; AOR: adjusted odds ratio; P: P-value; LWCI: lower chest wall indrawing; WBC: white blood cell

## Discussion

Our study investigated the magnitude and factors associated with bacteremia among children under five years of age diagnosed with CAP. We found a prevalence of bacteremia of 7.2% which was lower than that reported elsewhere in African countries [13-16]. The low prevalence of bacteremia in this setting may be partly due to the introduction and wide coverage of pneumococcal conjugate and *H. influenzae* type B vaccines, incidences of viral pneumonia, or the unreported use of antibiotics prior to obtaining blood cultures [17].

We observed a predominance of *S. aureus* among the isolates from positive blood cultures, which is comparable to findings from previous studies [14,18]. Trends and proportions of CAP etiology differ, largely owing to vaccination coverage. In Tanzania, for example, where diphtheria, tetanus, pertussis, hepatitis-b, and Hib (DTP-HepB-Hib) and Pneumococcal Conjugate Vaccine (PCV) coverage is more than 80% [19,20]. The principal cause of CAP (*H. influenzae* type b and pneumococcus) is expected to be relatively controlled through immunization. Moreover, the distribution among other known CAP aetiologies could be multifactorial [21-23]. Our findings suggest that bacterial species not targeted by current vaccines may dominate the CAP etiology, highlighting the need for continued surveillance and potential adjustments to vaccine strategies.

Of concern, the isolates were insensitive to amoxicillin and cephalosporin, WHO-recommended as first-line treatment for CAP in children. Our findings are in line with the emergence of antimicrobial drug resistance, particularly among isolates linked to CAP [24,25]. Owing to the challenges in accurately diagnosing and identifying the etiology of CAP, the WHO recommends empirical treatment for CAP in children [7]. This approach, along with prevalent antibiotic use malpractices, may have significantly contributed to the emergence of multidrug-resistant isolates associated with CAP in children.

We found that a positive blood culture could be predicted by fever, oxygen saturation, the need for oxygen therapy, a high respiratory rate, and leukocytosis. These clinical parameters would not be pathophysiologic signatures specific for blood culture positivity but rather have been associated with childhood pneumonia [26-29]. On the other hand, for instance, an increased number of WBCs is generally indicative of infection. In our study, leukocytosis (WBC >15,000/μl) was strongly associated with positive blood cultures, which is also consistent with findings from other studies [30,31]. Notably, neither age nor nutritional status of participants was associated with bacteraemia.

**Study limitation:** our study was limited to conventional blood culture, which may have been associated with inherent low sensitivity for isolate detection. Thus, we might have been unable to capture the entire spectrum of bacteremia among children with CAP, especially the attribution of fastidious microorganisms. Furthermore, the cross-sectional design of the study did not allow for follow-up of participants, thereby limiting our ability to assess outcomes such as the duration of hospitalization or compare lengths of stay between children with and without bacteraemia. These limitations should be considered when interpreting the findings and highlighting areas for further investigation.

## Conclusion

Taken together, our study revealed a predominance of *S. aureus* among positive blood cultures among children clinically diagnosed with CAP, whereas the majority of isolates exhibited relative resistance to antimicrobials, which are the WHO-recommended first-line treatments. These findings underscore the need for routine culture and susceptibility tests for effective CAP treatment and curbing the increasing burden of antimicrobial resistance.

### What is known about this topic

- Community-acquired pneumonia contributes a significant proportion of deaths among children under five in lower-middle-income countries, including Tanzania;
- There is a rising trend of multidrug-resistant organisms in bacterial isolates associated with CAP.

### What this study adds

- This study provides new insights into the distribution of bacterial isolates associated with bacteremia in children with CAP in our settings, highlighting the predominance of S. aureus species;
- We are reporting high resistance rates to WHO-recommended first-line antibiotics for pediatric CAP;
- The study highlights two key clinical predictors of bacteremia in children with CAP in the study settings being fever, oxygen saturation, the need for oxygen therapy, a high respiratory rate, and leucocytosis.

## Available online at

https://www.panafrican-med-journal.com//content/article/52/145/full
